# Effects of Physical Exercise on Endothelial Function and DNA Methylation

**DOI:** 10.3390/ijerph16142530

**Published:** 2019-07-16

**Authors:** Luca Ferrari, Marco Vicenzi, Letizia Tarantini, Francesco Barretta, Silvia Sironi, Andrea A. Baccarelli, Marco Guazzi, Valentina Bollati

**Affiliations:** 1 EPIGET, Epidemiology, Epigenetics and Toxicology Lab, Department of Clinical Sciences and Community Health, University of Milan, via san Barnaba 8, 20122 Milan, Italy; 2 Department of Cardiology, Fondazione IRCSS Ca’ Granda, Ospedale Maggiore Policlinico, Department of Clinical Sciences and Community Health, University of Milan, via san Barnaba 8, I-20122 Milan, Italy; 3 Unit of Clinical Epidemiology and Trial Organization, Fondazione IRCCS Istituto Nazionale dei Tumori, I-20133 Milan, Italy; 4 Department of Environmental Health Sciences, Columbia University Mailman School of Public Health, New York, NY 10032, USA; 5 Cardiology University Department, Heart Failure Unit, University of Milan, IRCCS Policlinico San Donato, San Donato Milanese, I-20097 Milan, Italy

**Keywords:** physical exercise, hypertension, DNA methylation, pyrosequencing, cardiovascular disease

## Abstract

Essential hypertension is the leading preventable cause of death in the world. Epidemiological studies have shown that physical training can reduce blood pressure (BP), both in hypertensive and healthy individuals. Increasing evidence is emerging that DNA methylation is involved in alteration of the phenotype and of vascular function in response to environmental stimuli. We evaluated repetitive element and gene-specific DNA methylation in peripheral blood leukocytes of 68 volunteers, taken before (T0) and after (T1) a three-month intervention protocol of continuative aerobic physical exercise. DNA methylation was assessed by bisulfite-PCR and pyrosequencing. Comparing T0 and T1 measurements, we found an increase in oxygen consumption at peak of exercise (VO_2peak_) and a decrease in diastolic BP at rest. Exercise increased the levels of ALU and Long Interspersed Nuclear Element 1 (LINE-1) repetitive elements methylation, and of Endothelin-1 (*EDN1*), Inducible Nitric Oxide Synthase (*NOS2*), and Tumour Necrosis Factor Alpha (*TNF*) gene-specific methylation. VO_2peak_ was positively associated with methylation of ALU, *EDN1*, *NOS2*, and *TNF*; systolic BP at rest was inversely associated with LINE-1, *EDN1*, and *NOS2* methylation; diastolic BP was inversely associated with *EDN1* and *NOS2* methylation. Our findings suggest a possible role of DNA methylation for lowering systemic BP induced by the continuative aerobic physical training program.

## 1. Introduction

Essential hypertension (EH) is a major public health concern and the leading preventable cause of premature death in many countries, due to its high prevalence and its association with coronary heart disease, stroke, renal disease, and other chronic disorders [1,2,3].

The association between EH and cardiovascular disease is supported by several studies [4,5,6]. Patients with blood pressure ≥ 180/110 mmHg have a greater risk of developing coronary heart disease than those with blood pressures ≤ 120/80 mmHg [7,8].

It has been estimated that by 2025 1.5 billion people will be affected by EH [9], resulting in a tremendous impact in terms of public health. Therefore, the World Health Organization (WHO) recommended a 25% relative reduction of the prevalence of hypertension among the public health targets to be achieved within 2020 in order to reduce the global burden of diseases [10].

EH is regarded as a multifactorial condition, the aetiology and severity of which are influenced by dynamic interactions among genetic and environmental factors [11,12]. It has been demonstrated that lifestyle modifications, including increased physical activity, can significantly contribute to blood pressure control [13,14]. A sedentary lifestyle increases the risk of many adverse health conditions, including cardiovascular diseases, and shortens life expectancy [15,16,17], whereas increased occupational or leisure-time physical activity is associated with lower levels of blood pressure [18,19,20]. A number of intervention studies have been conducted so far, supporting the role of physical activity in modulating blood pressure [21,22,23].

Physical training reduces blood pressure improving endothelial function pressure in hypertensive patients as well as in healthy individuals [24,25,26]. The mechanism of improvement in endothelial function during exercise has not been fully clarified, but it is thought that regular aerobic exercise reduces cellular reactive oxygen species (ROS) and increases nitric oxide (NO) bioavailability [27].

Environmental exposure and lifestyle factors impact on the genome by inducing epigenetic modifications and therefore making epigenetic determinants important susceptibility factors involved in complex multifactorial diseases, such as EH [28,29]. Interestingly, it was reported that conditions influenced by epigenetics, such as EH, can be reversed simply by making different lifestyle choices [30,31]. In addition, we reported, in a prospective study of aging conducted in the Boston area, that levels of leukocyte long interspersed nuclear element 1 (LINE-1) methylation predict future risks of ischemic heart disease and stroke [32].

The aim of the present study was to evaluate the effects of a three-month controlled aerobic physical training on DNA methylation, in hypertensive and healthy subjects. In particular, we measured DNA methylation in repetitive elements (ALU and LINE-1 sequences) as well as in specific genes chosen for the important role they have in inflammation and hypertension development processes: endothelial nitric oxide synthase (*NOS3*), endothelin 1 (*EDN1*), intercellular adhesion molecule 1 (*ICAM1*), inducible nitric oxide synthase (*NOS2*), toll-like receptor 2 (*TLR2*) and tumour necrosis factor alpha (*TNF*). Our research could provide important biological bases, as well as informative molecular markers, for assisting in a critical interpretation of clinical studies that suggest aerobic physical activity is potentially able to reduce risk and incidence of cardiovascular events in the general population.

## 2. Materials and Methods

### 2.1. Patients and Study Design

Subjects participating in this study were recruited from the Hypertension Outpatients Clinic at San Paolo Hospital, Milano, Italy. Forty-four patients with Essential Hypertension were enrolled at the time 0 (T0) among adults, immediately after the diagnosis or in the contest of a sub-optimal pharmacological treatment, and agreed to participate to an aerobic exercise training. Twenty-four normotensive healthy subjects had also been enrolled and were asked to follow the same exercise-training protocol of patients. The eligibility criteria for participants were: (1) being older than 18 years at enrolment; (2) being younger than 70 years at enrolment; (3) agreement to sign an informed consent and donate a blood sample. Exclusion criteria included: previous diagnosis of cancer, heart disease or stroke in the last year or other chronic diseases such as multiple sclerosis, Alzheimer’s disease, Parkinson’s disease, depression, bipolar disorder, schizophrenia, and epilepsy. Each study subject was asked to sign a consent form explaining the study aims and procedures and Institutional board approval was obtained (109/2012, Ethical Committee of Azienda Ospedaliera San Paolo, Milan).

The procedure for the first visit at baseline included: clinical examination, measurements of height, weight, systolic and diastolic blood pressure (respectively, SBP and DBP), information on the objectives and modalities of the study, signing of informed consent form, drawing of 13 mL of blood, to determine fasting blood glucose, lipid profile, liver function, creatine phosphokinase (CPK), renal function, electrolytes, complete blood count (CBC), and to extract DNA. A cardiopulmonary exercise testing (CPET) was performed in order to determine the functional capacity of each subject, to assess the oxygen consumption at peak of exercise (VO_2peak_, expressed in mL/kg/min and in % compared to a predicted maximum). Systemic blood pressure measurements were performed every 2 min and 12-lead continuous electrocardiogram (EKG) monitoring was recorded for the entire duration of CPET. All subjects were invited to limit lifestyle changes (diet, alcohol, smoke habit, hours of nightly sleep) during the 3-month training period except for the assigned physical activity program.

### 2.2. Exercise-Training Program

After the T0 visit, the study subjects started an aerobic exercise-training program four times per week, for a continuous 12 weeks. The protocol requested to perform at least 4 physical activity training sessions of 40 min a week (exercise on a stationary bike or jogging), in order to reach and maintain for at least 30 min the heart rate corresponding to the anaerobic threshold (AT), determined at the enrolment (T0). We considered uncompliant those subjects performing exercise training less than 4 times per week.

After 12 weeks (T1), a first follow-up visit was conducted. Evaluation of clinical parameters and biological sample collection was repeated, as in T0.

At the baseline and at the end of the exercise-training period, each subject underwent a symptom-limited CPET on a cycle ergometer. VO_2peak_, systolic and diastolic blood pressure were considered in the statistical analysis.

### 2.3. Sample Collection and DNA Methylation Analysis

We used ethylendiaminetetracetic acid (EDTA) tubes to collect 7 mL whole blood that was immediately frozen at −80 °C. DNA was extracted from whole blood using the Wizard Genomic DNA purification kit (Promega, Madison, WI, USA) following the manufacturer’s instructions.

We performed DNA methylation analyses on bisulphite-treated DNA using highly quantitative analysis based on PCR pyrosequencing; 500 ng DNA (concentration 25 ng/μL) was treated using the EZ DNA Methylation-Gold Kit (Zymo Research, Orange, CA, USA) according to the manufacturer’s protocol. Final elution was performed with 200 μL M-Elution Buffer.

To estimate repetitive element methylation, we performed DNA methylation analyses of ALU and LINE-1 repeated sequences, which allow for the amplification of a representative pool of repetitive elements, as previously described [33] Measures of ALU and LINE-1 methylation have been shown to be highly correlated with 5-methylcytosine content measured through high-performance liquid chromatography and are commonly used as a surrogate of global methylation [34,35].

Primers and PCR conditions for ALU, LINE-1, *NOS2*, *NOS3*, *ICAM1*, *TLR2*, *EDN1*, and *TNF* assays are shown in Supplementary Table S1. For each sequence, a 50 μL PCR was carried out in 25 μL GoTaq Green Master mix (Promega Corporation, Madison, WI, USA), 10 pmol forward primer, 10 pmol reverse primer, 50 ng bisulfite-treated genomic DNA, and water. PCR products were purified and sequenced by pyrosequencing as previously described [33] using 0.3 μM sequencing primer.

### 2.4. Statistical Analysis

Measurements were expressed as the mean + SD. Differences between variables at rest and after the peak exercise were tested for significance using two-sided Student’s t-test for paired data. Unadjusted and multivariable regression models (Adjusted for gender, age, BMI and smoking status as current/non-current) were applied to estimate the effects of a 1% increase in DNA methylation on cardiovascular outcomes (Peak VO_2_/Kg, Diastolic Blood Pressure—DBP- and Systolic Blood Pressure—SBP- at rest). All data management and statistical analyses were performed with SAS software, version 9.4 (SAS Institute Inc; Cary, NC, USA).

## 3. Results

### 3.1. Characteristics of the Study Participants

As summarized in Table 1, among the 68 study participants, 50 (73.5%) were males and 18 (26.5%) were females. The mean age was 49.5 years (range: 22–70 years). Considering the Body Mass Index (BMI) 26 (38.8%) subjects were classified as normal (BMI < 25), 32 (47.8%) subjects as overweight (BMI > 25 and < 30) and 9 (13.4%) subjects as obese (BMI ≥ 30).

Nine participants (13.2%) were current smokers and reported a median number of 15 cigarettes smoked every day (range between 0 and 30). For Blood Pressure, the participants showed wide ranges of values: 24 (35.3%) were classified as normal (diastolic blood pressure ≤ 80 and systolic blood pressure ≤ 120), 14 (20.6%) showed a border-line to mild hypertension (80 mmHg < diastolic blood pressure ≤ 90 mmHg and 120 mmHg < systolic blood pressure ≤ 140 mmHg) and 30 (44.1%) showed moderate hypertension (diastolic blood pressure > 90 and/or systolic blood pressure > 140).

### 3.2. Exercise-Induced Clinical Parameter Changes

Comparing at baseline and after 3 months of physical aerobic training protocol the clinical parameters we considered were changed (Table 2).

In particular, we observed an increase in VO_2peak_ from T0 to T1 (mean_T0_ = 24.8 mL/min/kg, SD = 10.0; mean_T1_ = 27.7 mL/min/kg, SD = 10.2; *p* < 0.0001) and a decrease in SBP at rest (mean_T0_ = 128.9 mmHg, mean_T1_ = 125 mmHg; *p* = 0.0026) and DBP at rest (mean_T0_ = 85.1 mmHg; mean_T1_ = 81.1 mmHg; *p* = 0.0001).

### 3.3. Exercise-Induced DNA Methylation Changes

In parallel with the changes observed in clinical outcomes, the comparison between T0 and T1 showed the methylation in two of the six genes and in both repetitive elements as significantly changed (Table 3).

Repetitive element methylation was increased in T1 compared to T0. In particular, ALU methylation showed a significant increase after training compared to baseline (mean_T0_ = 28.9%, SD = 4.6; mean_T1_ = 27.5%, SD = 4.4; *p* = 0.007), such as LINE-1 methylation (mean_T0_ = 78.2%, SD = 3.0; mean_T1_ = 79.8%, SD = 2.2; *p* = 0.001).

As we focused on gene-specific methylation, *EDN1* and *NOS2* showed higher methylation at the baseline (respectively, mean = 1.6, SD = 0.5; mean = 67.5, SD = 7.9) than after training (respectively, mean = 2.0, SD = 1.0; mean = 71.8, SD = 5.8) (*p* = 0.005 and *p* = 0.001). *NOS3*, *ICAM1*, *TLR2* and *TNF* showed a moderate non-significant increase in methylation (*p* > 0.1).

### 3.4. Association between Blood Pressure and Methylation Markers

Considering all the participants, methylation markers showed several significant associations with the VO_2peak_, as well as with SBP and DBP (Table 4).

In particular, when we considered a multivariate model adjusted for gender, age, body mass index, and smoking status, VO_2peak_ was significantly associated to ALU methylation (*β* = 0.38, *p* = 0.004), *EDN1* methylation (*β* = 0.93, *p* = 0.046), *NOS2* methylation (*β* = 0.16, *p* = 0.009) and *TNF* (*β* = 0.14, *p* = 0.042).

SBP at rest was inversely associated with methylation of LINE-1 (*β* = −0.90, *p* = 0.023), *EDN1* (*β* = −3.0, *p* = 0.003), and *NOS2* (*β* = −0.40, *p* = 0.004) and DBP at rest was inversely associated with methylation of *EDN1* (*β* = −1.7, *p* = 0.035), and *NOS2* (*β* = −0.3, *p* = 0.008).

## 4. Discussion

In the present study, we evaluated the effects of a continuative aerobic physical training on pressure parameters and on methylation levels of ALU and LINE-1 repetitive elements and of six specific genes (*NOS3, EDN1, NOS2, ICAM1, TLR2, TNF*) measured on leukocyte DNA.

The cardiovascular efficacy of the exercise-training period is demonstrated by the increase of VO_2peak_ and the lowering of both DBP and SBP. Our data confirm literature data regarding the protective role of physical activity on cardiovascular risk factors, such as lower levels of LDL cholesterol and blood markers of inflammation, reduced blood pressure, decreased insulin resistance and reduction of body mass index [36,37,38,39].

Our results showed that a continuative aerobic physical training is able to modify DNA methylation in leukocytes. This finding is suggestive of a more generalized effect of physical training, as several studies showed that DNA methylation levels measured in blood closely correlates with methylation patterns of other tissues and therefore provides a proxy for methylation patterns in other tissues [6,40,41]. DNA methylation levels showed also associations with blood pressure values. These findings, taken together, are suggestive of a possible role exerted by DNA methylation in the complex molecular mechanism that links physical training to changes in cardiovascular performances.

Accumulating evidence suggests that regular aerobic physical activity could reduce the risk and incidence of cardiovascular ischemic events in the general population [42]. In particular, physical activity may be able to confer cardiovascular protection through a direct effect on vessel walls. Physical activity has been associated with reduced risks of all-cause mortality [43,44] and it has an important role in preventing cardiovascular diseases [45,46,47], modulating oxidative stress and inflammation [48].

The relationship between epigenetic modifications and cardiovascular disease is well consolidated [49,50,51]. In addition, recent studies suggested that physical exercise is able to alter epigenetic modifications involved in cardiovascular risk [42,52].

We found a negative association between LINE-1 methylation and systolic pressure and a negative association between both systolic and diastolic pressure and *EDN1* and *NOS2* methylation.

The results obtained regarding repetitive element methylation are consistent with previous studies showing a negative association between cardiovascular diseases and LINE-1 methylation [32,53,54,55,56]. In particular, leukocyte hypomethylation of repetitive sequences, such as LINE-1, have been associated with prevalent ischemic heart disease and stroke in the Normative Aging Study conducted in the Boston area [32]. In addition, LINE-1 hypomethylation has previously been associated with altered levels of LDL and HDL [57].

Interestingly, a negative association was also reported between global DNA methylation levels and the severity of EH [40].

Endothelin- 1 (encoded by *EDN1* gene) is a potent arterial vasoconstrictor and an elevated plasma level is a prognostic marker in patients with cardiovascular diseases as well as a predictor of mortality in patients with cardiovascular diseases [58]. Therefore, reduction of *EDN1* methylation levels may be involved in mediating the effects of both systolic and diastolic pressure increase.

In mammals, the production of NO is catalyzed by three isoforms of NOS encoded by separate genes mapped on three different chromosomes: neuronal NOS (*NOS1*), inducible NOS (*NOS2*) and endothelial NOS (*NOS3*).

Immune activation mediated by pro-inflammatory cytokines is associated with the expression of *NOS2* and the production of NO [59]. Specific studies on *NOS2* gene have shown that lower DNA methylation in the *NOS2* promoter is associated with increased *NOS2* expression [60]. While *NOS3* is constitutively expressed and responsible for the majority of NO produced by the vascular endothelium, *NOS2* expression, and its activity are increased in the presence of reactive oxygen species (ROS) [61]. We previously reported that DBP was positively associated with *NOS2* methylation levels in Peripheral blood mononuclear cells (PBMCs) in the elderly population of the Normative Aging Study (NAS) [62]. However, several confounders may be considered, such as the age of the subjects, diet, previous cardiovascular events. These factors might explain the different results obtained in different populations, such as the one described in this paper and the NAS population, supporting the complex effects of epigenetic modifications.

Epigenetics provides a newer vantage point for understanding transcriptional control paradigms in NO synthases. In particular, these pathways may represent the molecular substrate for the modulation of cardiovascular disease susceptibility.

The present study must be interpreted taking into account both strengths and limitations. First, the sample size is limited. Therefore, the results of this study should be considered as preliminary and we recommend future studies to confirm our findings. However, all study participants were recruited in the same hospital and clinical investigations were standardized as they were performed by cardiologists after a training program and using the same instrumentation.

Second, we selected LINE-1, ALU, *NOS3, EDN1, NOS2, ICAM1, TLR2, TNF* for DNA methylation analysis for their role in inflammatory pathways and cardiovascular disease and because they are expressed in many human tissues, including blood, the source of DNA for our study. However, several other genes might have been included in our study. Future studies investigating a larger number of target genes, possibly in an unbiased way, should be conducted.

Third, the quite large variation in the difference between methylation at baseline and after training suggests that not only physical activity but also other factors might have a role, such as unchangeable or non-depending individual factors (such as environmental and genetic factors).

Moreover, the study population was characterized by a wide range of blood pressure measures, giving us the possibility to evaluate the association of DNA methylation and blood pressure in subjects characterized by variable levels of disease.

Finally, blood DNA is derived from a mixed cell population and we cannot exclude that differences in the number of subpopulations might have contributed to determine the results we observed in the present work.

Taken as a whole these factors might contribute to the variability in DNA methylation response and define individual phenotypes.

## 5. Conclusions

Our findings provide a rationale to support a role for DNA methylation in mediating the protective effect of physical training on cardiovascular risk. If these results shall be confirmed, individual measures of DNA methylation might be useful to assess the beneficial effect of physical activity and to predict the reduction of cardiovascular risk.

## Figures and Tables

**Table 1:** Characteristics of the Study Participants at the Baseline (T0).

	All Subjects	Diastolic Blood Pressure ≤ 80 and Systolic Blood Pressure ≤ 120	80< Diastolic Blood Pressure ≤ 90 and 120 < Systolic Blood Pressure ≤ 140	Diastolic Blood Pressure > 90 and/or Systolic Blood Pressure > 140
	(*n* = 68)	(*n* = 24, 35.3%)	(*n* = 14, 20.6%)	(*n* = 30, 44.1%)
**Gender, *n* (%)**				
Male	50 (73.5)	17 (25.0)	9 (13.2)	24 (35.3)
Female	18 (26.5)	7 (10.3)	5 (7.4)	6 (8.8)
**Age (Years), *n* (%)**				
22–40	17 (25.0)	4 (5.9)	4 (5.9)	9 (13.2)
41–60	33 (48.5)	12 (17.6)	4 (5.9)	17 (25.0)
61–70	18 (26.5)	8 (11.8)	5 (7.35)	5 (7.35)
**BMI (kg/m^2^), *n* (%)**				
≤25	26 (38.8)	13 (19.4)	5 (7.5)	8 (11.9)
>25 and ≤30	32 (47.8)	10 (14.9)	5 (7.5)	17 (25.4)
>30	9 (13.4)	1 (1.5)	3 (4.5)	5 (7.4)
**Smoking, *n* (%)**				
Never smoker	59 (86.8)	22 (32.3)	11 (16.2)	26 (38.3)
Current smoker	9 (13.2)	2 (2.9)	3 (4.4)	4 (5.9)
**Compliance (Physical Training) ^a^, *n* (%)**				
Yes	52 (76.5)	19 (27.9)	10 (14.7)	23 (33.8)
No	16 (23.5)	5 (7.3)	4 (5.9)	7 (10.3)

**^a^** Compliance was based on the completion at least four complete physical activity training sessions of 40 min a week (exercise on a stationary bike or jogging), in order to reach and maintain for at least 30 min the heart rate corresponding to the anaerobic threshold (AT), determined at the enrolment (T0). BMI: body mass index.

**Table 2:** Cardiovascular outcomes at Baseline (T0) and after Physical Training (T1).

Methylation Marker	Baseline (T0)					Post Physical Training (T1)					*p*-Value
	Mean	SD	25pct	Median	75pct	Mean	SD	25pct	Median	75pct	
Peak VO_2_/Kg (mL min^-1^ Kg^-1^)	24.8	8.4	18.9	22.9	30.7	27.7	8.5	22.0	25.9	32.4	<0.0001
SBP at rest (mmHg)	128.9	13.8	120.0	130.0	135.0	125.0	12.4	117.5	120.0	132.5	0.0026
DBP at rest (mmHg)	85.1	12.0	80.0	82.5	90.0	81.1	10.0	75.0	80.0	85.0	0.0001

^a^ Paired *t*-test. SBP: systolic blood pressure; DBP: diastolic blood pressure.

**Table 3:** Methylation Level at Baseline (T0) and after Physical Training (T1).

Methylation Marker	*n*	Baseline (T0)					Post Physical Training (T1)					*p*-Value ^a^
		Mean	SD	25pct	Median	75pct	Mean	SD	25pct	Median	75pct	
ALU	67	27.5	4.4	24.4	25.6	34.1	28.9	4.6	25.2	26.5	34.5	0.007
LINE-1	59	78.2	3.0	77.0	78.5	80.5	79.8	2.2	78.7	79.9	81.2	0.001
*NOS3*	61	81.1	9.0	78.4	84.1	86.2	82.8	8.2	82.5	84.8	86.5	0.277
*EDN1*	65	1.6	0.5	1.3	1.7	1.9	2.0	1.0	1.4	1.9	2.2	0.005
*NOS2*	65	67.5	7.9	61.5	68.2	74.5	71.8	5.8	66.9	72.5	76.6	0.001
*ICAM1*	64	6.7	2.3	4.9	6.0	8.2	7.2	3.1	5.3	6.4	8.1	0.321
*TLR2*	66	9.6	7.2	2.8	8.9	14.0	10.3	10.8	2.5	7.5	14.2	0.686
*TNF*	66	14.6	6.7	11.1	13.8	16.9	14.9	5.4	12.1	14.1	16.2	0.846

^a^ Paired *t*-test.

**Table 4:** Unadjusted and multivariable regression models (Adjusted for gender, age, BMI and smoking status) estimating the effects of a 1% increase in DNA methylation on cardiovascular outcomes (Peak VO_2_/Kg, PAS at rest and PAD at rest).

	*N*	Unadjusted Models			Multivariable Models		
		*β ^a^*	(95% CI)	*p*-Value	*β ^a^*	(95% CI)	*p*-Value
**Peak VO_2_/Kg (mL min^−1^ Kg^−1^)**							
ALU	134	0.3	(0.01, 0.6)	0.045	0.38	(0.13, 0.64)	0.004
LINE-1	118	0.37	(0.02, 0.72)	0.039	0.31	(−0.01, 0.63)	0.059
*NOS3*	122	0.08	(−0.07, 0.24)	0.299	0.04	(−0.1, 0.18)	0.553
*EDN1*	130	0.95	(−0.06, 1.96)	0.064	0.93	(0.02, 1.83)	0.046
*NOS2*	130	0.17	(0.03, 0.3)	0.014	0.16	(0.04, 0.27)	0.009
*ICAM1*	128	0.06	(−0.25, 0.37)	0.699	0.08	(−0.21, 0.36)	0.587
*TLR2*	132	0	(−0.1, 0.11)	0.935	0	(−0.11, 0.09)	0.823
*TNF*	132	0.16	(0.02, 0.31)	0.029	0.14	(0.01, 0.27)	0.042
**Systolic blood pressure at rest (mmHg)**							
ALU	134	−0.4	(−0.92, 0.14)	0.146	−0.5	(−1.02, 0.05)	0.074
LINE-1	118	−0.9	(−1.6, −0.14)	0.021	−0.9	(−1.59, −0.12)	0.023
*NOS3*	122	−0.1	(−0.36, 0.21)	0.582	0	(−0.31, 0.26)	0.854
*EDN1*	130	−3.0	(−4.93, −0.99)	0.004	−3.0	(−4.89, −1.02)	0.003
*NOS2*	130	−0.4	(−0.63, −0.1)	0.007	−0.4	(−0.65, −0.13)	0.004
*ICAM1*	128	−0.1	(−0.73, 0.52)	0.740	−0.2	(−0.86, 0.4)	0.474
*TLR2*	132	0.1	(−0.11, 0.32)	0.344	0.11	(−0.11, 0.32)	0.323
*TNF*	132	0	(−0.31, 0.29)	0.940	0.01	(−0.29, 0.31)	0.957
**Diastolic blood pressure at rest (mmHg)**							
ALU	134	−0.4	(−0.81, 0.08)	0.104	−0.3	(−0.77, 0.1)	0.125
LINE-1	118	−0.6	(−1.19, 0.06)	0.074	−0.5	(−1.11, 0.12)	0.112
*NOS3*	122	−0.2	(−0.42, 0.05)	0.125	−0.1	(−0.34, 0.12)	0.328
*EDN1*	130	−2.0	(−3.62, −0.29)	0.022	−1.7	(−3.34, −0.12)	0.035
*NOS2*	130	−0.3	(−0.51, −0.07)	0.012	−0.3	(−0.5, −0.08)	0.008
*ICAM1*	128	−0.1	(−0.65, 0.41)	0.654	−0.2	(−0.7, 0.35)	0.506
*TLR2*	132	0.07	(−0.11, 0.24)	0.465	0.05	(−0.12, 0.22)	0.555
*TNF*	132	−0.1	(−0.36, 0.14)	0.378	−0.1	(−0.34, 0.15)	0.459

^a^ To estimate the effects of DNA methylation on cardiovascular outcomes, the level of each sequence methylation was examined in relation to cardiovascular outcomes. This association was verified on all the measures performed in the study, regardless of whether they were measured on samples taken at baseline (i.e., T0) or after physical training (T1). In the models, the two samples collected at different times are exchangeable, thus assuming that DNA methylation levels produced similar modifications at the two time points.

## Supplementary Materials

The following are available online at https://www.mdpi.com/1660-4601/16/14/2530/s1, Supplementary Table S1: Primer sequences and PCR conditions.
