# Supplementary material for: Effects of Physical Exercise on Endothelial Function and DNA Methylation
Source: Int J Environ Res Public Health. 2019 Jul 16;16(14):2530. doi: 10.3390/ijerph16142530 (PMC6678332; doi:10.3390/ijerph16142530)
Supplement: Supplementary file 1 [file ijerph-16-02530-s001.pdf]

Table 1: Primer sequences and PCR conditions.

Sequence ID	Forward primer (5' to 3')	Reverse primer (5' to 3')	Sequencing primer (5' to 3')	PCR conditions
**GLOBAL DNA METHYLATION MARKERS**				
**ALU**	biotin-TTTTTATT AAAAATATAAAA ATT	CCCAAACTAAAA TACAATAA	AATAACTAAAAT TACAAAC	96°C for 90'' 43°C for 60'' 72°C for 120'' (40 cycles)
**LINE-1**	TTTTGAGTTAGG TGTGGGATA TA	biotin-AAAATCAA AAAATTCCCTTTC	AGTTAGGTGTG GGATATAGT	95°C for 30'' 50°C for 30'' 72°C for 30'' (35 cycles)
**GENE-SPECIFIC METHYLATION MARKERS**				
**eNOS**	TGTAGTTTTAGGGTT TTGTTGGA	biotin-CCCCTATCCCATAC AAT	TATTAGTTTTAGT TTTTATA	95°C for 1' 58°C for 30'' 72°C for 1' (50 cycles)
**Et-1**	TTGTTTGGGGTTGG AATAAAGT	biotin-ATCCTCAACCCAAA TACCCTTTT	GGTAGAGAGTTG TTTAAGTT	95°C for 1' 56°C for 1' 72°C for 1' (50 cycles)
**iNOS**	AATGAGAGTTGTTG GGAAGTGTTT	biotin-CCACCAAACCCAA CCAAACT	TAAAGGTATTTTT GTTTTAA	95°C for 1' 60°C for 1' 72°C for 1' (45 cycles)
**ICAM**	biotin-TGAGGGGTATTTTTG ATGTTTGT	ACTAAAAAATACCC CTCCCCCTAA	TCCACACCTAAC AC	95°C for 1' 56°C for 1' 72°C for 1' (45 cycles)
**TLR2**	GTTTTTTTTTGATTT GGAATTTT	biotin-TTCCAAAC AAATAACCCC	GGAGTTTGTTGG GAAGTA	95°C for 1' 54,5°C for 1' 72°C for 1' (45 cycles)
**TNF-alpha**	biotin-TGAGGGGTATTTTTG ATGTTTGT	CCAACAACTACCTT TATATATCCC	ATAAACCCTACAC CTCTAT	95°C for 1' 57°C for 1' 72°C for 1' (50 cycles)
